# Supplementary material for: Redescription of Phymolepis cuifengshanensis (Antiarcha: Yunnanolepididae) using high-resolution computed tomography and new insights into anatomical details of the endocranium in antiarchs
Source: PeerJ. 2018 May 28;6:e4808. doi: 10.7717/peerj.4808 (PMC5978403; doi:10.7717/peerj.4808)
Supplement: Supplemental Information 1 — Phylogenetic analysis: (1) Character list. (2) Taxa and principal sources of data. (3) Data matrix with 79 morphological characters for 44 taxa. (4) Characters and character states defining major clades shown in Fig. S1. (5) Supplementary references. [file peerj-06-4808-s001.docx]

Supplementary Text for

**Redescription of *Phymolepis cuifengshanensis* (Antiarcha: Yunnanolepididae) using high-resolution computed tomography and new insights into anatomical details of the endocranium in antiarchs**

Yajing Wang^1, 2, 3^ and Min Zhu^1, 2, 3^

^1^Key Laboratory of Vertebrate Evolution and Human Origins of Chinese Academy of Sciences, Institute of Vertebrate Paleontology and Paleoanthropology, Chinese Academy of Sciences, Beijing 100044, China

^2^CAS Center for Excellence in Life and Paleoenvironment, Beijing, 100044, China

**Phylogenetic Analysis**

**1) Character list**

**Ornamentation, histology and scales**

1. **Adult ornamentation**:

tubercular (0);

reticular (1).

Zhang and Young (1992, Character 0); Zhu (1996, Character 33); Jia, Zhu & Zhao (2010, Character 46); Pan et al. (2017, Character 33).

1. **Adult ornamentation:**

non-ridged (0);

ridged (1).

Zhang and Young (1992, Character 1); Zhu (1996, Character 34, part); Jia, Zhu & Zhao (2010, Character 47, part); Pan et al. (2017, Character 34, part).

1. **Ridges on dorsal wall of trunk shield:**

converging (0);

subparallel (1).

Zhang and Young (1992, Character 9); Zhu (1996, Character 34, part); Jia, Zhu & Zhao (2010, Character 48, part); Pan et al. (2017, Character 34, part).

1. **Dorsal spongy layer in dermal bone of trunk shield:**

absent (0);

present (1).

Young (1984, 1988, Character 34); Zhu (1996, Character 36); Pan et al. (2017, Character 36).

1. **Ridged scales:**

absent (0);

present (1).

Zhu (1996, Character 35); Pan et al. (2017, Character 35).

**Head shield and neurocranium**

1. **Premedian plate:**

absent (0);

present (1).

Young (1984, 1988, Character 7); Zhu (1996, Character 44); Pan et al. (2017, Character 44).

1. **Premedian plate:**

short and broad (0);

long and narrow (1).

Zhu (1996, Character 51); Jia, Zhu & Zhao (2010, Character 1); Pan et al. (2017, Character 51).

1. **Anterior margin of premedian plate:**

convex (0);

slightly concave (1).

Zhu (1996, Character 52); Jia, Zhu & Zhao (2010, Character 2); Pan et al. (2017, Character 52).

1. **Unornamented shelf and rostrocaudal groove on premedian plate:**

absent (0);

present (1).

Young (1984, 1988, Character 36); Zhu (1996, Character 53); Jia, Zhu & Zhao (2010, Character 3); Pan et al. (2017, Character 53).

1. **Rostral width/orbital width index of premedian plate:**

smaller than 200 (0).

larger than 200 (1).

Zhu (1996, Character 54); Jia, Zhu & Zhao (2010, Character 4); Pan et al. (2017, Character 54).

1. **Lateral plate:**

absent (0);

present (1).

Young (1984, 1988, Character 3); Zhu (1996, Character 43); Pan et al. (2017, Character 43).

1. **Lateral plate:**

narrow (0);

broad (1).

Zhu (1996, Character 50); Jia, Zhu & Zhao (2010, Character 5); Pan et al. (2017, Character 50).

1. **Preorbital depression:**

present (0);

absent (1).

Zhang and Young (1992, Character 11); Zhu (1996, Character 45); Jia, Zhu & Zhao (2010, Character 6); Pan et al. (2017, Character 45).

1. **Preorbital depression:**

extending laterally onto lateral plates (0);

restricted to premedian plate (1).

Zhu (1996, Character 46); Jia, Zhu & Zhao (2010, Character 7); Pan et al. (2017, Character 46).

1. **Preorbital recess:**

absent (0);

present (1);

Young (1984, 1988, Character 25); Zhang and Young (1992, Character 11); Zhu (1996, Character 47, part); Jia, Zhu & Zhao (2010, Character 8, part); Pan et al. (2017, Character 47, part).

1. **Preorbital recess:**

restricted to premedian plate (0);

extending laterally to the lateral plates (1).

Zhu (1996, Character 47, part); Jia, Zhu & Zhao (2010, Character 8, part); Pan et al. (2017, Character 47, part).

1. **Orbital opening:**

open (0);

enclosed by skull roof plates (1).

Zhu (1996, Character 48); Pan et al. (2017, Character 48).

1. **Orbital fenestra:**

large (0);

small (1).

Zhu (1996, Character 55); Jia, Zhu & Zhao (2010, Character 10); Pan et al. (2017, Character 55).

1. **Relative position of orbital fenestra (ordered):**

anterior (0);

slightly anterior (1);

slightly posterior (2);

posterior (3).

Zhu (1996, Character 56); Jia, Zhu & Zhao (2010, Character 11); Pan et al. (2017, Character 56).

1. **Nasal opening:**

at anterolateral corners of rostral plate (0);

at anterior margin of rostral plate (1).

Young (1984, 1988, Character 35); Zhu (1996, Character 49); Jia, Zhu & Zhao (2010, Character 9); Pan et al. (2017, Character 49).

1. **Postpineal and nuchal plates:**

long and narrow (0);

short and broad (1).

Zhu (1996, Character 57); Jia, Zhu & Zhao (2010, Character 12); Pan et al. (2017, Character 57).

1. **Pronounced postpineal thickening:**

absent (0);

present (1).

Young (1984, 1988, Character 38); Zhu (1996, Character 62); Jia, Zhu & Zhao (2010, Character 13); Pan et al. (2017, Character 62).

The term ‘postpineal thickening’ used in phylogenetic analysis was named after Young (1983). This term refers to the thickening on the anterior margin of the postorbital crista in the visceral surface of postpineal plate.

1. **Position of postorbital crista:**

extending medially to postpineal plate (0);

extending obliquely to nuchal plate (1).

New character.

1. **Nuchal plate:**

without orbital facets (0).

with orbital facets (1).

Young (1984, 1988, Character 26); Zhang and Young (1992, Character 16); Zhu (1996, Character 58); Jia, Zhu & Zhao (2010, Character 14); Pan et al. (2017, Character 58).

1. **Supraotic thickening of head shield:**

absent (0);

present (1).

New character.

1. **Median occipital crista of head shield:**

absent (0);

present (1).

New character.

The median occipital crista, also termed as the posterior median process of head shield (Hemmings 1978), is positioned on the visceral surface head shield. It differs from the posterior process of head shield (Miles 1968; Young 1988).

1. **Posterior process of head shield:**

absent (0);

present (1).

New character.

1. **Obstantic margin:**

long (0);

short (1).

Young (1984, 1988, Character 28); Zhu (1996, Character 59); Jia, Zhu & Zhao (2010, Character 15); Pan et al. (2017, Character 59).

1. **Central sensory canal:**

present (0);

absent (1).

Young (1984, 1988, Character 24); Zhang and Young (1992, Character 6); Zhu (1996, Character 37); Jia, Zhu & Zhao (2010, Character 20); Pan et al. (2017, Character 37).

1. **Supraorbital canal:**

present (0);

absent (1).

Zhu (1996, Character 38); Pan et al. (2017, Character 38).

1. **X-shaped pit-line grooves:**

present (0);

absent (1).

Zhu (1996, Character 39); Jia, Zhu & Zhao (2010, Character 21); Pan et al. (2017, Character 39).

1. **Branch of infraorbital canal diverging on lateral plate:**

present (0);

absent (1).

Zhu (1996, Character 40); Jia, Zhu & Zhao (2010, Character 22); Pan et al. (2017, Character 40).

1. **Semicircular pit-line:**

absent (0);

present (1).

Zhu (1996, Character 41); Jia, Zhu & Zhao (2010, Character 23); Pan et al. (2017, Character 41).

1. **Occipital cross-commissure issued from infraorbital canal:**

absent or short (0);

long and extending onto nuchal plate (1).

Young (1984, 1988, Character 19, part); Zhu (1996, Character 42); Jia, Zhu & Zhao (2010, Character 24); Pan et al. (2017, Character 42).

1. **Anterior postorbital process:**

behind anterior level of orbital notch (0);

extending in front of orbital notch (1).

Young (1984, 1988, Character 37); Zhang and Young (1992, Character 17); Zhu (1996, Character 61); Jia, Zhu & Zhao (2010, Character 17); Pan et al. (2017, Character 61).

We term the “endocranium postorbital process” as the “anterior postorbital process” and re-formulate state 0 (“short”) as “behind anterior level of orbital fenestra”.

1. **Anterior postorbital process:**

at or behind posterior level of orbital notch (0);

in front of posterior level of orbital notch (1).

New character.

1. **Cavity for cranio-spinal process:**

absent (0);

present (1).

New character.

1. **Supraoccipital pit of head shield:**

absent (0);

present (1).

New character.

This pit or cavity, also termed as the supraotic pit (Long & Werdelin 1986), corresponds to the endocranial supraoccipital process.

1. **Confluence between anterior and posterior semicircular canals:**

midway between orbital notch and transverse nuchal crista or close to orbital notch (0);

close to transverse nuchal crista (1).

New character.

1. **Endolymphatic duct through head shield:**

long tube (0);

short tube (1).

New character.

1. **Occipital portion of endocranium**

long (0);

short (1).

New character.

1. **Submarginal articulation:**

absent (0);

present (1).

Zhang and Young (1992, Character 13); Zhu (1996, Character 60); Jia, Zhu & Zhao (2010, Character 16); Pan et al. (2017, Character 60).

1. **Postsuborbital plate:**

present (0);

absent (1).

Zhu (1996, Character 63); Jia, Zhu & Zhao (2010, Character 18); Pan et al. (2017, Character 63).

The plate was termed as the prelateral plate in Zhu (1996).

1. **Postsuborbital plate:**

with a long anterior process (0);

equilateral, triangular in shape (1).

Zhang and Young (1992, Character14); Zhu (1996, Character 64); Pan et al. (2017, Character 64).

1. **Postsuborbital plate:**

behind suborbital plate (0):

above suborbital plate (1).

Zhu (1996, Character 65); Pan et al. (2017, Character 65).

The suborbital was termed as the mental plate in Zhu (1996).

1. **Suborbital plates of both sides:**

separated (0):

meeting in the midline (1).

Young (1984, 1988, Character 32); Zhu (1996, Character 66); Jia, Zhu & Zhao (2010, Character 19); Pan et al. (2017, Character 66).

**Trunk shield**

1. **Shape of trunk shield:**

low and elongated (0);

high and short (1).

Young (1984, 1988, Character 33); Zhu (1996, Character 10); Pan et al. (2017, Character 10); Jia, Zhu & Zhao (2010, Character 25);

1. **Number of median dorsal plates:**

one (0);

two (1).

Young (1984, 1988, Character 1); Zhu (1996, Character 11); Pan et al. (2017, Character 11).

1. **Index (r_1_) between width of anterior margin and maximum width of anterior median dorsal plate (ordered):**

r1>55 (0);

35=<r1=<55 (1);

15=<r1<35 (2);

<15 (3).

Young (1984, 1988, Character 22); Zhang and Young (1992, Character 3); Zhu (1996, Character 12); Jia, Zhu & Zhao (2010, Character 33); Pan et al. (2017, Character 12).

The anterior median dorsal plate of antiarchs is homologous to the median dorsal plate 1 in *Qilinyu* (Zhu et al. 2016) and the extrascapular plate in *Entelognathus* (Zhu et al. 2013), and some arthrodires (Goujet 1973). It is absent in *Kujdanowaspis*.

1. **Index (r_2_) between anterior and posterior divisions of anterior median dorsal plate (ordered):**

r_2_<300 (0);

300=<r_2_=<500 (1);

>500 (2).

Zhu (1996, Character 13); Pan et al. (2017, Character 13).

1. **Tergal angle of anterior median dorsal plate:**

centrally or posteriorly placed (0);

anteriorly placed (1).

Zhang and Young (1992, Character 2); Zhu (1996, Character 14); Jia, Zhu & Zhao (2010, Character 34); Pan et al. (2017, Character 14).

This tergal angle roughly corresponds to the anterior ventral pit in position.

1. **Dorsal spine of anterior median dorsal plate:**

absent (0);

present (1).

Zhu (1996, Character 19); Jia, Zhu & Zhao (2010, Character 39); Pan et al. (2017, Character 19).

1. **Anterior median dorsal plate.**

completely overlapping anterior dorsolateral plate (0);

partly overlapping anterior dorsolateral plate (1).

Zhu (1996, Character 15); Jia, Zhu & Zhao (2010, Character 35); Pan et al. (2017, Character 15).

1. **Anterior median dorsal plate:**

underlapping or partly overlapping posterior dorsolateral (or mixilateral) plate (0);

completely overlapping posterior dorsolateral (or mixilateral) plate (1).

Young (1984, 1988, Character 17); Zhu (1996, Character 16); Jia, Zhu & Zhao (2010, Character 36); Pan et al. (2017, Character 16).

1. **Anterior median dorsal plate.**

partly or completely overlapping posterior dorsolateral (or mixilateral) plate (0);

underlapping posterior dorsolateral (or mixilateral) plate (1).

Young (1984, 1988, Character 21); Zhang and Young (1992, Character 4); Zhu (1996, Character 17); Jia, Zhu & Zhao (2010, Character 37); Pan et al. (2017, Character 17).

1. **Anterior ventral process and pit on anterior median dorsal plate:**

present (0);

absent (1).

Young (1984, 1988, Character 31); Zhu (1996, Character 18); Jia, Zhu & Zhao (2010, Character 38); Pan et al. (2017, Character 18).

1. **Lateral process of posterior median dorsal plate:**

conspicuous (0);

reduced (1).

Young (1984, 1988, Character 16); Zhu (1996, Character 20); Pan et al. (2017, Character 20).

1. **Crista transversalis interna posterior of trunk shield:**

lying laterally to posterior ventral pit and process of posterior median dorsal plate (0).

lying behind posterior ventral pit and process of posterior median dorsal plate (1).

Zhu (1996, Character 21); Jia, Zhu & Zhao (2010, Character 40); Pan et al. (2017, Character 21).

1. **Posterior ventral pit and process of posterior median dorsal plate:**

on crista transversalis interna posterior (0);

posteriorly migrated behind crista transversalis interna posterior (1).

Young (1984, 1988, Character 14); Zhu (1996, Character 22); Pan et al. (2017, Character 22).

1. ***Crista transversalis interna posterior* of trunk shield:**

lying laterally to posterior ventral process and pit (0).

turning anteriorly and in front of posterior ventral process and pit (1).

Zhu (1996, Character 23); Pan et al. (2017, Character 23).

1. **Anterior lateral plate:**

present (0);

absent (1).

Young (1984, 1988, Character 2); Zhu (1996, Character 24); Pan et al. (2017, Character 24).

1. **Chang’s apparatus:**

absent (0);

present (1).

Zhu (1996, Character 25); Pan et al. (2017, Character 25).

1. **Ventrolateral fossa of trunk shield:**

absent (0);

present (1).

Zhu (1996, Character 26); Pan et al. (2017, Character 26).

1. **Posterior dorsolateral and posterior lateral plates:**

independent (0);

fused to form a mixilateral plate (1).

Young (1984, 1988, Character 20); Zhang and Young (1992, Character 8); Zhu (1996, Character 27); Jia, Zhu & Zhao (2010, Character 41); Pan et al. (2017, Character 27).

1. **Posterior ventrolateral and posterior lateral plates:**

independent (0);

fused to form (or replaced by) a single plate (1).

Young (1984, 1988, Character 13); Zhang and Young (1992, Character 7); Zhu (1996, Character 28); Jia, Zhu & Zhao (2010, Character 42); Pan et al. (2017, Character 28).

1. **Semilunar plate:**

paired (0);

unpaired (1).

Young (1984, 1988, Character 23); Zhang and Young (1992, Character 5); Zhu (1996, Character 29); Jia, Zhu & Zhao (2010, Character 44); Pan et al. (2017, Character 29).

1. **Large rectangular aperture on ventral wall of trunk shield:**

absent (0);

present (1).

Young (1984, 1988, Character 15); Zhu (1996, Character 30); Jia, Zhu & Zhao (2010, Character 45); Pan et al. (2017, Character 30).

1. **Spinal plate:**

present (0);

absent (1).

Young (1984, 1988, Character 11, part); Zhu (1996, Character 31); Pan et al. (2017, Character 31).

1. **Postbranchial lamina:**

external and upright (0);

internal and horizontal (1).

Zhu (1996, Character 32); Pan et al. (2017, Character 32).

1. **Pectoral fin:**

scale-covered (0);

modified into a slender appendage covered with small dermal plates (1).

Young (1984, 1988, Character 8); Zhu (1996, Character 1); Pan et al. (2017, Character 1).

1. **Number of plates encircling pectoral fenestra:**

two or more (0);

one (1).

Zhu (1996, Character 2); Pan et al. (2017, Character 2).

1. **Brachial process:**

absent (0);

present (1);

Young (1984, 1988, Character 9); Zhu (1996, Character 3, part); Pan et al. (2017, Character 3, part).

1. **Brachial process:**

simple (0);

helmet-shaped (1);

Young (1984, 1988, Character 9); Zhu (1996, Character 3, part); Jia, Zhu & Zhao (2010, Character 31); Pan et al. (2017, Character 3, part).

1. **Axillary foramen:**

small (0);

large (1).

Young (1984, 1988, Character 27); Zhang and Young (1992, Character 15); Zhu (1996, Character 4); Jia, Zhu & Zhao (2010, Character 32); Pan et al. (2017, Character 4).

1. **Pectoral appendage:**

unjointed (0);

jointed (1).

Young (1984, 1988, Character 10); Zhu (1996, Character 5); Jia, Zhu & Zhao (2010, Character 27); Pan et al. (2017, Character 5).

1. **Dorsal central plate 1 and dorsal central plate 2 of pectoral appendage:**

in contact (0);

separated (1).

Zhang and Young (1992, Character 18); Zhu (1996, Character 6); Jia, Zhu & Zhao (2010, Character 28); Pan et al. (2017, Character 6).

1. **Pectoral appendage:**

short (0);

elongated (1).

Zhang and Young (1992, Character 12); Zhu (1996, Character 7); Jia, Zhu & Zhao (2010, Character 26); Pan et al. (2017, Character 7).

1. **Lateral marginal plate 2 relative to trunk shield:**

short (0);

elongated (1).

Zhu (1996, Character 8); Jia, Zhu & Zhao (2010, Character 29); Pan et al. (2017, Character 8).

1. **Number of lateral marginal plates of distal segment:**

three (0);

two (1).

Zhu (1996, Character 9); Jia, Zhu & Zhao (2010, Character 30); Pan et al. (2017, Character 9).

**2) Taxa and principal sources of data**

**Outgroup (2 OTUs)**

*Kujdanowiaspis*: Stensiö, 1945; Stensiö, 1963; Goujet, 1984; Dupret, Goujet & Mark-Kurik, 2007; Dupret, 2010.

*Romundina*: Ørvig, 1975; Goujet & Young 2004; Dupret et al., 2014; Dupret et al., 2017.

**Ingroup (42 OTUs)**

*Vanchienolepis*: Tông-Dzuy & Janvier, 1990.

*Chuchinolepis:* Chang, 1978; Zhang, 1978; Zhang, 1984; Tông-Dzuy & Janvier, 1990; Liu, 1992; Zhu, Wang & Fan, 1994.

*Heteroyunnanolepis:* Wang, 1994; Zhu, 1996.

*Zhanjilepis:* Zhang, 1978; Liu, 1992; Zhu, 1996.

*Yunnanolepis:* Liu, 1963; Chang, 1978; Zhang, 1978; Zhang, 1980b; Liu, 1992; Zhu, Wang & Fan, 1994; Zhu, 1996.

*Mizia*: Zhu, 1996.

*Phymolepis*: Chang, 1978; Zhang, 1978; Zhu, 1996.

*Parayunnanolepis*: Zhang, Wang & Wang, 2001; Zhu et al., 2012.

*Minicrania*: Zhu & Janvier, 1996.

*Liujiangolepis*: Wang, 1987.

*Dayaoshania*: Ritchie et al., 1992.

*Xichonolepis*: P'an & Wang, 1978; Zhang, 1980a; Ritchie et al., 1992.

*Grenfellaspis*: Ritchie et al., 1992.

*Sinolepis*: Liu & P'an, 1958; Pan et al., 1987; Ritchie et al., 1992.

*Luquanolepis*: Zhang & Young, 1992.

*Nawagiaspis*: Young, 1990.

*Dianolepis*: Chang, 1965.

*Wudinolepis*: Chang, 1965.

*Microbrachius*: Hemmings, 1978; Pan, 1984; Wang & Zhang, 1999; Long et al., 2015.

*Hohsienolepis*: P'an et al., 1978.

*Wufengshania*: Pan et al., 2017.

*Grossilepis*: Gross, 1941a; Gross, 1942a; Stensiö, 1948; Lukševičs, 2001; Olive, 2015.

*Bothriolepis*: Gross, 1942b; Stensiö, 1948; Chang, 1965; Miles, 1968; Liu, 1973; Long, 1983; Young, 1984; Long & Werdelin, 1986; Young, 1988; Johanson, 1997a; Lukševičs, 2001; Moloshnikov, 2008; Moloshnikov, 2011; Olive, 2015; Pan et al., 1980.

*Monarolepis*: Young & Gorter, 1981; Young, 1988; Long et al., 1990.

*Briagalepis*: Long et al., 1990.

*Tenizolepis*: Malinovskaya, 1992; Moloshnikov, 2011.

*Vietnamaspis*: Long et al., 1990.

*Jiangxilepis*: Zhang & Liu, 1991.

*Ningxialepis*: Jia, Zhu & Zhao, 2010.

*Sherbonaspis*: Young & Gorter, 1981.

*Wurungulepis*: Young, 1990.

*Pteryichthyodes*: Hemmings, 1978.

*Hunnanolepis*: P'an et al., 1978.

*Kirgisolepis*: Panteleyev, 1992.

*Byssacanthus*: Woodward, 1895; Karatajūte-Talimaa, 1960.

*Gerdalepis*: Gross, 1941b; Friman & Koln, 1982.

*Lepadolepis*: White & Moy-Thomas, 1940; Denison, 1978.

*Grossaspis*: White & Moy-Thomas, 1940.

*Stegolepis*: Malinovskaya, 1973.

*Asterolepis*: Lyarskaya, 1977; Moloshnikov, 2008; Olive, 2015.

*Pambulaspis*: Young, 1983.

*Remigolepis*: Andrews, 1978; Pan et al., 1980; Pan et al. 1987; Johanson, 1997b; Moloshnikov, 2008; Olive, 2015.

**3) Data matrix with 79 morphological characters for 44 taxa**

? = unavailable character; - = logical impossibility. Data are interleaved to facilitate copying and pasting into NEXUS format.

*Kujdanowiaspis* 00-000----0-?-??000???-000100000000000000-010000--------?00000000000000--------

*Romundina* 00-00100000-0-0-000000-000110000000000000-??0011?-------?00000000-10000--------

*Chuchinolepis*  00-0?1000011000-110?00?000001000000??1???0????013010000000001??000011110-00000-

*Vanchienolepis*  00-0??????????????????????????????????????????01301000010???1??000111110-1????-

*Zhanjilepis* 00-0?????????????????0????????????????????????013000000000001?100?0???????????-

*Heteroyunnanolepis* 00-0010000111??0110?00?0?000?00??0???????0????013000000000001??000011110-0????-

*Yunnanolepis*  00-0?1000011000-110?0000000010000000110000????013000000000011110000?1110-00000-

*Mizia*  00-0?1000011000-110000?0??0011000000?????0000?01300000000001111000011110-00000-

*Phymolepis*  00-0??0???11???-11??00000000110?000011000?????01300000000001111000011110-00000-

*Parayunnanolepis* 00-001000011000-100?00?0??00110?000??????0????0130000000000111?000011110-00000-

*Minicrania*  00-0?10000100010100?00?0??0010110100?10??0????010010000000001??000011110-0?????

*Liujiangolepis*  00-00100011100??112?10?0???011110000?????0????010010010000?01000101111110010100

*Xichonolepis*  00-0?100011101??111000?0???0111100???????0????012110010000101000101111110010?00

*Dayaoshania* 00-0?100011101??110?10?0???0111100???????0????012110010000??1000101111110010000

*Grenfellaspis*  00-0?10001110110110?00?00000111100???1?110????011210010010101000101111110010?00

*Sinolepis* 00-0?100011001??110?00?0?00011110000?????0????010210010010??1000101111110010?00

*Wudinolepis*  0100?110001100??103?00?????001110101?01???????01001000100100100011011111111?01?

*Hohsienolepis*  0110?110001100??103000?0???001110?????????????010010001001001000110111111?1?0??

*Microbrachius*  0110?1100011000-103?0000011001110101001111????01001000100100100011011111111100?

*Bothriolepis*  10-0011000111-11112010111110010010110111110110010010001001001000110111111111111

*Grossilepis*  00-0?11000111-11112?10?1?1100100101100???1011?010010010001001000110111111111111

*Wufengshania* 00-0010000111-11102?10111?1101000001001111???????????????????????????1111??????

*Briagalepis*  00-0????????????1??????????????00????????1????010010101001001000110111111??????

*Monarolepis* 00-0????????????1???????111?0?1??????0111?????010010101001001000110111111011?1?

*Vietnamaspis*  00-00???????1-??1?????????????????????????????011010001011001?0???011111111???1

*Dianolepis* 00-0?11000111-11102?10?0???001010001??1??1????0110100010010010001101111110101??

*Tenizolepis* 00-0?11000111-1110201??0???011?10?????????????010010101001001000110111111?1??1?

*Luquanolepis* 00-0????????????1?????????????????????????????0110100??00100100001011111101?01?

*Nawagiaspis* 00-0?11000101-??103?10??????11??0001?????10010111010???00100100001011111101????

*Jiangxilepis* 10-0?11000101-11102?1000?0?01111000100111?????01100100110100100101011111111011?

*Ningxialepis* 10-001????1?????1??????0???00?0??1???????0????111001101?011010010?01?1111?1011?

*Kirgisolepis* 00-0?11000101-10102?1??0???1010011????????1???111001101?010010010?011111111????

*Hunanolepis*  00-0?11000101-10103010?011?1111101010011101???111000001101001001000111111010011

*Wurungulepis* 00-01???????????1?????????????????????????????11?000001?010010000001111110?????

*Sherbonaspis* 00-0?10100101-1010??10?0??11111??101?????01???112000001101001001000111111010000

*Stegolepis* 0?-0?11100101-10101?1??0???1111101????????1???11200100010100100000011111101000?

*Byssacanthus* 00-0?11000101-10102?00?0???1111111???????01???111001000101001000000111111010001

*Pterichthyodes*  00-0111000101-10102?10101111110101010011101??1111000001101001001000111111010000

*Grossaspis* 00-1????????????1?????????????????????????????11100000110100100?0?01111110?????

*Lepadolepis* 00-1????????????1?????????????????????????????11100000110100100?0?011111101000?

*Gerdalepis* 00-1?10000101-1010000??0???1111101????????1???111000001101001001010111111010000

*Pambulaspi*s 00-0?10110101-101011110011111111010100111?1???0130000011010010000001111110?000?

*Asterolepis* 00-0110110101-10101110001011111101010011101??1013000010101001001000111111010001

*Remigolepis* 00-0?10110101-1010111100101111110101?011101??1013000000101001000000?1111100000-

**4) Characters and character states defining major clades shown in Supplementary Figure 1**

Asterisks stand for ambiguous character transformation resolved using DELTRAN and ACCTRAN. Character state is “1”, unless marked otherwise.

*Delayed-transformation apomorphy lists (DELTRAN):*

Node 1 (Antiarcha): 11, 17, 27(0), 29, 38, 61, 68, 69, 70, 71;

Node 2 (Yunnanolepidoidei): 49(3)*;

Node 3: 18*;

Node 4: 51(0), 63*;

Node 6: 37*, 60, 62*;

Node 7: 30;

Node 9 (Minicrania + Sinolepididae + Euantiarcha): 15, 31, 32;

Node 10 (Sinolepididae + Euantiarcha): 30, 40*, 41*, 65, 72, 75*;

Node 11 (Sinolepididae): 10, 18, 54, 67;

Node 12: 14, 50, 59*;

Node 13: 49(2);

Node 14: 50(2), 57;

Node 15 (Euantiarcha): 7, 19(2)*, 26, 27, 36, 38(0), 39*, 42, 55, 58, 66, 73*;

Node 16 (Microbrachiidae): 2, 19(3), 29(0);

Node 17: 34*, 74*;

Node 18: 13, 21, 25, 45*, 78*, 79*;

Node 19: 16;

Node 20: 29(0), 31(0)*, 77*;

Node 21: 23*, 24, 32(0), 76;

Node 22: 74;

Node 24: 53;

Node 26 (Asterolepidoidei): 49, 65(0);

Node 27: 12(0)*, 47;

Node 28: 42(0), 43, 51(0), 56, 64;

Node 29: 52, 74, 77;

Node 30: 29(0), 31(0), 34*, 53;

Node 31: 28, 34*, 66(0);

Node 32: 5*, 46*, 78(0);

Node 33: 64;

Node 34: 55(0);

Node 35: 8, 19, 49(2);

Node 36: 7(0), 9, 20*, 47(0), 49(3);

Node 37: 26(0)

Node 38: 79(0);

Node 39: 7(0);

Node 40: 4;

*Accelerated-transformation apomorphy lists (ACCTRAN):*

Node 1 (Antiarcha): 11, 17, 27(0), 29, 38, 61, 68, 69, 70, 71;

Node 2 (Yunnanolepidoidei): 18*, 37*, 49(3)*, 62*, 63*;

Node 4: 51(0);

Node 5: 13*;

Node 6: 60;

Node 7: 30;

Node 9 (Minicrania + Sinolepididae + Euantiarcha): 15, 31, 32, 40, 41, 45, 75;

Node 10 (Sinolepididae + Euantiarcha): 19(2)*, 30, 39*, 41*, 65, 72;

Node 11 (Sinolepididae): 10, 18, 54, 59*, 67;

Node 12: 14, 19(0)*, 49*, 50;

Node 13: 49(2);

Node 14: 50(2), 57;

Node 15 (Euantiarcha): 7, 26, 27, 36, 38(0), 42, 55, 58, 66, 73*, 79*;

Node 16 (Microbrachiidae): 2, 15(0)*, 19(3), 29(0), 74*, 76*;

Node 18: 13, 21, 25;

Node 19: 16, 23*, 31(0)*, 44*, 77*;

Node 20: 29(0);

Node 21: 24, 32(0), 76;

Node 22: 18*, 33*, 35*, 74;

Node 24: 7(0)*, 28*, 53;

Node 26 (Asterolepidoidei): 12(0)*, 49, 65(0);

Node 27: 47;

Node 28: 34*, 42(0), 43, 51(0), 56, 64;

Node 29: 1*, 26(0)*, 52, 74, 77;

Node 30: 29(0), 31(0), 32(0)*, 33*, 53;

Node 31: 5*, 28, 34*, 66(0);

Node 32: 78(0);

Node 33: 20*, 64;

Node 34: 52*, 55(0);

Node 35: 8, 19, 22*, 49(2);

Node 36: 7(0), 9, 20*, 47(0), 49(3);

Node 37: 26(0)

Node 38: 23*, 79(0);

Node 39: 7(0), 19(0);

Node 40: 4, 21(0)*, 66*;

**6) Supplementary references**

Andrews S. 1978. A possible occurrence of *Remigolepis* in the topmost Old Red Sandstone of Berwickshire. *Scottish Journal of Geology* 14:311-315.

Chang K-J. 1965. New antiarchs from the Middle Devonian of Yunnan. *Vertebrata PalAsiatica* 9:1-9.

Chang K-J. 1978. Early Devonian antiarchs from Cuifengshan, Yunnan. In: Institute of Geology and Mineral Resources of the Chinese Academy of Geological Sciences, ed. *Symposium on the Devonian System of South China*. Beijing: Geological Press, 292-297.

Denison RH. 1978. Placodermi. In: Schultze H-P, ed. Handbook of Paleoichthyology, vol 2. Stuttgart: Gustav Fischer Verlag: 128.

Dupret V. 2010. Revision of the genus *Kujdanowiaspis* Stensiö, 1942 (Placodermi, Arthrodira, “Actinolepida”) from the Lower Devonian of Podolia (Ukraine). *Geodiversitas* 32:5-63. DOI: 10.5252/g2010n1a1.

Dupret V, Goujet D, Mark-Kurik E. 2007. A new genus of placoderm (Arthrodira: 'Actinolepida') from the Lower Devonian of Podolia (Ukraine). *Journal of Vertebrate Paleontology* 27:266-284.

Dupret V, Sanchez S, Goujet D, Ahlberg PE. 2017. The internal cranial anatomy of Romundina stellina Orvig, 1975 (Vertebrata, Placodermi, Acanthothoraci) and the origin of jawed vertebrates-Anatomical atlas of a primitive gnathostome. *PLoS One* 12:e0171241. DOI: 10.1371/journal.pone.0171241.

Dupret V, Sanchez S, Goujet D, Tafforeau P, Ahlberg PE. 2014. A primitive placoderm sheds light on the origin of the jawed vertebrate face. *Nature* 507:500-503. DOI: 10.1038/nature12980.

Friman L, Koln. 1982. Ein mitteldevonischer Antiarche, Gerdalepis jesseni n. sp., aus der SoStenicher Mulde, Nord-Eifel (Rheinisches Schiefergebirge). *Paläontologische Zeitschrift* 56:229-234.

Goujet DF. 1973. *Sigaspis*, un nouvel arthrodire du Dévonien inférieur du Spitsberg. *Palaeontographica Abteilung A* 143:73-88.

Goujet DF. 1984. *Les poissons placodermes du Spitsberg. Arthrodires Dolichothoraci de la Formation de Wood Bay (Dévonien inférieur)*. Paris Editions Centre National Recherche Scientifique, Cahiers de Paléontologie.

Goujet DF, Young GC. 2004. Placoderm anatomy and phylogeny: new insights. In: Arratia G, Wilson MVH, Cloutier R, eds. *Recent Advances in the Origin and Early Radiation of Vertebrates*. München: Verlag Dr. Friedrich Pfeil, 109-126.

Gross W. 1941a. Die *Bothriolepis*-Arten der Cellulosa-Mergel Lettlands. *Kungliga Svenska Vetenskapsakademiens Handlingar* 19:1-79.

Gross W. 1941b. Neue Beobachtungen an *Gerdalepis rhenana* (Beyrich). *Palaeontographica Abt A* 93.

Gross W. 1942a. Die Fischfaunen des baltischen Devons und ihre biostratigraphische Bedeutung. *Korrespondenz-blatt des Naturforscher-Vereins zu Riga* 64:373-436.

Gross W. 1942b. Über Knochen-Mißbildungen bei Asterolepiden. *Paläontologische Zeitschrift* 23:206-218.

Hemmings SK. 1978. The Old Red Sandstone antiarchs of Scotland: *Pterichthyodes* and *Microbrachius*. *Monographs of the Palaeontographical Society* 131:1-64.

Jia L-T, Zhu M, Zhao W-J. 2010. A new antiarch fish from the Upper Devonian Zhongning Formation of Ningxia, China. *Palaeoworld* 19:136-145. DOI: 10.1016/j.palwor.2010.02.002.

Johanson Z. 1997a. New antiarchs (Placodermi) from the Hunter Siltstone (Famennian) near Grenfell, N.S.W. *Alcheringa* 21:191-217. DOI: 10.1080/03115519708619173.

Johanson Z. 1997b. New *Remigolepis* (Placodermi; Antiarchi) from Canowindra, New South Wales, Australia. *Geological Magazine* 134:813-846.

Karatajūte-Talimaa VN. 1960. *Byssacanthus dilatatus* (Eichw.) from the Middle Devonian of the USSR. *Collectanea Acta Geologica Lithuanica*: 293-305.

Liu S-F. 1973. New materials of *Bothriolepis shaokuanensis* and the age of the fish bearing beds. *Vertebrata PalAsiatica* 11:37-42.

Liu S-F. 1992. On the Lower Devonian antiarchians of Guangxi, China. *Vertebrata PalAsiatica* 30:210-220.

Liu T-S, P'an K. 1958. Devonian fishes from Wutung Series near Nanking, China. *Palaeontologica Sinica, new series C* 141:1-41.

Liu Y-H. 1963. On the Antiarchi from Chutsing, Yunnan. *Vertebrata PalAsiatica* 7:39-46.

Long JA. 1983. New bothriolepid fish from the late Devonian of Victoria, Australia. *Palaeontology* 26:295-320.

Long JA, Burrett CF, Ngan PK, Janvier P. 1990. A new bothriolepid antiarch (Pisces, Placodermi) from the Devonian of Do Son peninsula, northern Vietnam. *Alcheringa* 14:181-194. DOI: 10.1080/03115519008619054.

Long JA, Mark-Kurik E, Johanson Z, Lee MS, Young GC, Zhu M, Ahlberg PE, Newman M, Jones R, Blaauwen JD, Choo B, Trinajstic K. 2015. Copulation in antiarch placoderms and the origin of gnathostome internal fertilization. *Nature* 517:196-199. DOI: 10.1038/nature13825.

Long JA, Werdelin L. 1986. A new Late Devonian bothriolepid (Placodermi, Antiarcha) from Victoria, with description of other species from the state. *Alcheringa* 10:355-399.

Lukševičs E. 2001. Bothriolepid antiarchs (Vertebrata, Placodermi) from the Devonian of the north-western part of the East European Platform. *Geodiversitas* 23:489-609.

Lyarskaya L. 1977. Noviye danniye o *Asterolepis ornata* Eichwald iz rannefranskih otlozheniy Pribaltiki. *Notes on Phylogeny and Systematic of Fossil Fish and Agnathans*. Moscow: Nauka publ., 36-44.

Malinovskaya S. 1973. A new Middle Devonian genus *Stegolepis* (Antiarchi, Placodermi) from central Kazakhstan.

Malinovskaya S. 1992. New Middle Devonian antiarchs (Placodermi) of Central Kazakhstan. In: Mark-Kurik E, ed. *Fossil Fishes as Living Animals*. Tallinn: Academy of Sciences of Estonia, 177-184.

Miles RS. 1968. The Old Red Sandstone antiarchs of Scotland: Family Bothriolepididae. *Palaeontographical Society Monographs* 122:1-130.

Moloshnikov SV. 2008. Devonian antiarchs (Pisces, Antiarchi) from central and Southern European Russia. *Paleontological Journal* 42:691-773. DOI: 10.1134/s0031030108070010.

Moloshnikov SV. 2011. Bothriolepiform antiarchs (Pisces, Placodermi) from the Devonian of Central Kazakhstan. *Paleontological Journal* 45:291-304. DOI: 10.1134/s0031030111030099.

Olive S. 2015. Devonian antiarch placoderms from Belgium revisited. *Acta Palaeontologica Polonica* 60:711-731.

Ørvig T. 1975. Description, with special reference to the dermal skeleton, of a new radotinid arthrodire from the Gedinnian of Arctic Canada. *Problèmes actuels de Paléontologie-Evolution des Vertébrés*. Paris: Colloques Internationaux du Centre National de la Recherche Scientifique, 41-71.

P'an K, Wang S-T. 1978. Devonian Agnatha and Pisces of South China. *Symposium on the Devonian System of South China*. Beijing: Geological Press, 298-333.

P'an K, Wang S-T, Gao L-D, Hou J-P. 1978. The Devonian System of South China. *Symposium on the Devonian System of South China*, 31.

Pan J. 1984. A new species of *Microbrachius* from Middle Devonian of Yunnan. *Vertebratea Palasiatica* 22:9-13.

Pan J, Huo F-C, Cao J-X, Gu Q-C, Liu S-Y, Wang J-Q, Gao L-D, Liu C. 1987. *Continental Devonian System of Ningxia and its Biotas*. Beijing: Geological Publishing House.

Pan J, Wang S-T, Liu S-Y, Gu Q-C, Jia H. 1980. Discovery of Devonian *Bothriolepis* and *Remigolepis* in Ningxia. *Acta Geologica Sinica* 3:175-185.

Pan Z-H, Zhu M, Zhu Y-A, Jia L-T. 2017. A new antiarch placoderm from the Emsian (Early Devonian) of Wuding, Yunnan, China. *Alcheringa*: 1-12. DOI: 10.1080/03115518.2017.1338357.

Panteleyev N. 1992. New remigolepids and high armoured antiarchs of Kirgizia. *Fossil Fishes as Living Animals*. Tallinn: Academy of Sciences of Estonia, 185-192.

Ritchie A, Wang S-T, Young GC, Zhang G-R. 1992. The Sinolepidae, a family of antiarchs (placoderm fishes) from the Devonian of South China and eastern Australia. *Records of the Australian Museum* 44:319-370. DOI: 10.3853/j.0067-1975.44.1992.38.

Stensiö EA. 1945. On the heads of certain arthrodires. 2. On the cranium and cervical joint of the Dolichothoraci. *Kungliga Svenska Vetenskapsakademiens Handlingar* 22:1-70.

Stensiö EA. 1948. On the Placodermi of the Upper Devonian of East Greenland. II. Antiarchi: subfamily Bothriolepinae. With an attempt at a revision of the previously described species of that family. *Meddelelser om Grønland* 139:1-622.

Stensiö EA. 1963. Anatomical studies on the arthrodiran head. Part 1. Preface, geological and geographical distribution, the organization of the head in the Dolichothoraci, Coccosteomorphi and Pachyosteomorphi. Taxonomic appendix. *Kungliga Svenska Vetenskapsakademiens Handlingar* 9:1-419.

Tông-Dzuy T, Janvier P. 1990. Les Vertébrés du Dévonien inférieur du Bac Bo oriental (provinces de Bac Thaï et Lang Son, Viêt Nam). *Bulletin du Muséum national d'Histoire naturelle, Paris 4e sér, Section C* 12:143 223.

Wang J-Q, Zhang G-R. 1999. New material of *Microbrachius* from lower Devonian of Qujing, Yunnan, China. *Vertebrata PalAsiatica* 37:200-211.

Wang S-T. 1987. A new antiarch from the Early Devonian of Guangxi. *Vertebratea Palasiatica* 25:81-90.

Wang Z-S. 1994. New discovery of yunnanolepids - *Heteroyunnanolepis qujingensis* gen. et sp. nov. *Vertebrata PalAsiatica* 32:11.

White E, Moy-Thomas J. 1940. Notes on the nomenclature of fossil fishes. Part I: Homonyms A-C. *Annals and Magazine of Natural History* 5:502-507.

Woodward AS. 1895. XVI.—On the Devonian Ichthyodorulite, *Byssacanthus*. *Journal of Natural History Series 6* 15:142-144. DOI: 10.1080/00222939508677860.

Young GC. 1983. A new antiarchan fish (Placodermi) from the Late Devonian of southeastern Australia. *BMR Journal of Australian Geology & Geophysics* 8:71-81.

Young GC. 1984. Reconstruction of the jaws and braincase in the Devonian placoderm fish *Bothriolepis*. *Palaeontology* 27:635-661.

Young GC. 1988. Antiarchs (Placoderm fishes) from the Devonian Aztec Silstone, Southern Victoria Land, Antarctica. *Palaeontographica Abt A* 202:1-125.

Young GC. 1990. New antiarchs (Devonian placoderm fishes) from Queensland, with comments on placoderm phylogeny and biogeography. *Memoirs of the Queensland Museum* 28:35-50.

Young GC, Gorter JD. 1981. A new fish fauna of Middle Devonian age from the Taemas/Wee Jasper region of New South Wales. *Bulletin of the Bureau of Mineral Resources Geology and Geophysics Australia* 209:85-147.

Zhang G-R. 1978. The antiarchs from the Early Devonian of Yunnan. *Vertebrata PalAsiatica* 16:147-186.

Zhang G-R. 1980a. New material of *Xichonolepis qujingensis* and discussion on some of its morphological characteristics. *Vertebrata PalAsiatica* 18:272-280.

Zhang G-R. 1984. New form of Antiarchi with primitive brachial process from Early Devonian of Yunnan. *Vertebrata PalAsiatica* 22:81-91.

Zhang G-R, Liu Y-G. 1991. A new antiarch from the Upper Devonian of Jiangxi, China. In: Chang M-M, Liu Y-H, Zhang G-R, eds. *Early Vertebrates and Related Problems of Evolutionary Biology*. Beijing: Science Press, 195-212.

Zhang G-R, Wang J-Q, Wang N-Z. 2001. The structure of pectoral fin and tail of Yunnanolepidoidei, with a discussion of the pectoral fin of chuchinolepids. *Vertebrata PalAsiatica* 39:9-19.

Zhang G-R, Young GC. 1992. A new antiarch (placoderm fish) from the Early Devonian of South China. *Alcheringa* 16:219-240.

Zhang M-M. 1980b. Preliminary note on a Lower Devonian antiarch from Yunnan, China. *Vertebrata PalAsiatica* 18:179-190.

Zhu M. 1996. The phylogeny of the Antiarcha (Placodermi, Pisces), with the description of Early Devonian antiarchs from Qujing, Yunnan, China. *Bulletin du Muséum national d'Histoire naturelle* 18:233-347.

Zhu M, Ahlberg PE, Pan Z-H, Zhu Y-A, Qiao T, Zhao W-J, Jia L-T, Lu J. 2016. A Silurian maxillate placoderm illuminates jaw evolution. *Science* 354:334-336. DOI: 10.1126/science.aah3764.

Zhu M, Janvier P. 1996. A small antiarch, *Minicrania lirouyii* gen. et sp. nov., from the Early Devonian of Qujing, Yunnan (China), with remarks on antiarch phylogeny. *Journal of Vertebrate Paleontology* 16:1-15.

Zhu M, Wang J-Q, Fan J-H. 1994. Early Devonian fishes from Guijiatun and Xujiachong Formations of Qujing, Yunnan, and related biostratigraphic problems. *Vertebrata PalAsiatica* 32:1-20.

Zhu M, Yu X-B, Ahlberg PE, Choo B, Lu J, Qiao T, Qu Q-M, Zhao W-J, Jia L-T, Blom H, Zhu Y-A. 2013. A Silurian placoderm with osteichthyan-like marginal jaw bones. *Nature* 502:188-193. DOI: 10.1038/nature12617.

Zhu M, Yu X-B, Choo B, Wang J-Q, Jia L-T. 2012. An antiarch placoderm shows that pelvic girdles arose at the root of jawed vertebrates. *Biology Letters* 8:453-456. DOI: 10.1098/rsbl.2011.1033.
