# Supplementary figures and images for: Redescription of Phymolepis cuifengshanensis (Antiarcha: Yunnanolepididae) using high-resolution computed tomography and new insights into anatomical details of the endocranium in antiarchs
Source: PeerJ. 2018 May 28;6:e4808. doi: 10.7717/peerj.4808 (PMC5978403; doi:10.7717/peerj.4808)

Weighted amount of change  
(Minimum)  
Bar shading: CI

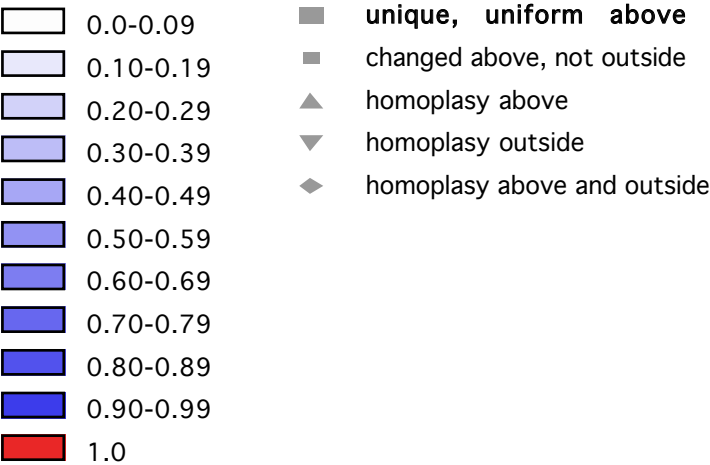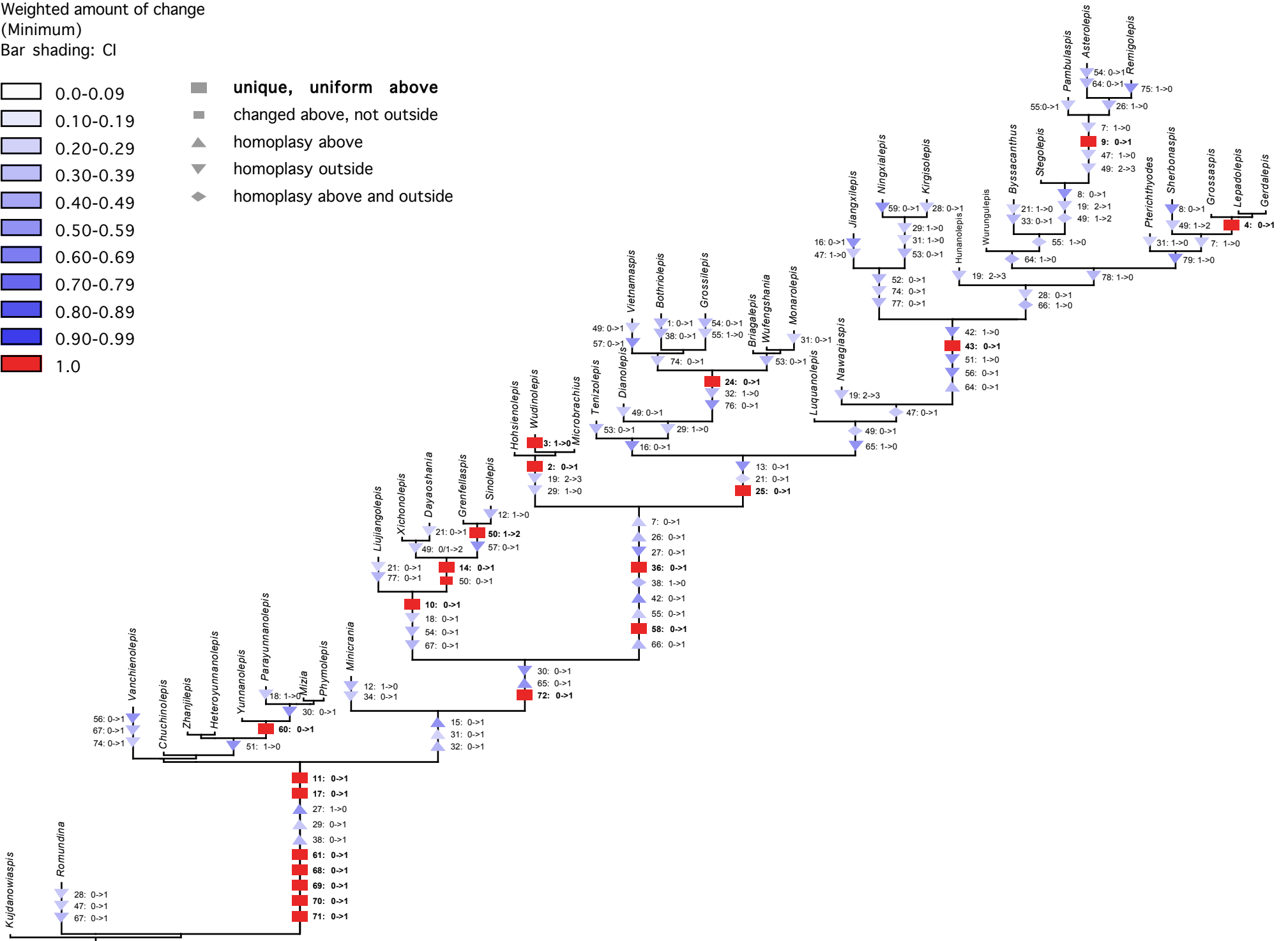

Supplement: Figure S1 — Unambiguous character state changes mapped across one of the most parsimonious trees (Fig. 14). [file peerj-06-4808-s003.pdf]
